# Dynamic knee control and movement strategies in athletes and non‐athletes in side hops: Implications for knee injury

**DOI:** 10.1111/sms.13432

**Published:** 2019-04-25

**Authors:** Jonas L. Markström, Helena Grip, Lina Schelin, Charlotte K. Häger

**Affiliations:** ^1^ Department of Community Medicine and Rehabilitation, Physiotherapy Umeå University Umeå Sweden; ^2^ Department of Radiation Sciences, Biomedical Engineering and Physics Umeå University Umeå Sweden; ^3^ Department of Statistics, Umeå School of Business, Economics and Statistics Umeå University Umeå Sweden

**Keywords:** biomechanics, injury prevention, kinematics, kinetics, sports

## Abstract

Athletes exposed to rapid maneuvers need a high level of dynamic knee stability and robustness, while also controlling whole body movement, to decrease the risk of non‐contact knee injury. The effects of high‐level athletic training on such measures of movement control have not, however, been thoroughly evaluated. This study investigated whether elite athletes (who regularly perform knee‐specific neuromuscular training) show greater dynamic knee robustness and/or different movement strategies than non‐athletic controls, in relation to overall knee function. Thirty‐nine women (19 athletes, 20 controls) performed standardized rebound side hops (SRSH) while a motion capture system synchronized with two force plates registered three‐dimensional trunk, hip, and knee joint angles and moments. Dynamic knee robustness was evaluated using finite helical axis (FHA) inclination angles extracted from knee rotation intervals of 10°, analyzed with independent *t* tests. Angle and moment curves were analyzed with inferential methods for functional data. Athletes had superior knee function (less laxity, greater hop performances, and strength) but presented similar FHA inclination angles to controls. Movement strategies during the landing phase differed; athletes presented larger (a) hip flexion angles (during 9%‐29% of the phase), (b) hip adduction moments (59%‐99%), (c) hip internal rotation moments (83%‐89%), and (d) knee flexion moments (79%‐93%). Thus, elite athletes may have a greater ability than non‐athletes to keep the knee robust while performing SRSH more efficiently through increased engagement of the hip. However, dynamic knee robustness associated with lower FHA inclination angles still show room for improvement, thus possibly decreasing knee injury risk.

## 1 INTRODUCTION

Dynamic knee stability has been defined as the ability to keep the knee joint stable when subjected to rapidly changing loads.^1^ More specifically, knee robustness has been defined as the ability to cope with uncertainties and disturbances, while the knee is considered stable until injury occurs.^2^ Dynamic knee stability is challenged in single‐leg landing tasks with side‐to‐side movement, which frequently occur in team sports, for example football, floorball, and basketball. Non‐contact anterior cruciate ligament (ACL) injury commonly occurs in such maneuvers with multi‐plane knee loading in weight‐bearing with the knee in a relatively straight, abducted, and rotated position.^3^, ^4^, ^5^ The ability to maintain dynamic knee stability in sport‐similar tasks is therefore of utmost importance among athletes. Such dynamic knee stability is maintained during dynamically challenging tasks when frontal and transversal plane motions are minimal.^6^, ^7^ These movements have indeed shown to decrease the strain placed on the ACL,^8^, ^9^, ^10^ with obvious implications for injury prevention.

There is, however, a general lack of objective measures and consensus on how to evaluate dynamic knee stability and robustness. Discrete kinematic and kinetic values are commonly employed, but these measures are mainly descriptive without relating to simultaneous motions in all three planes. It remains undetermined whether high athletic training leads to definite gains in dynamic knee robustness or altered task execution. Since the combination of knee frontal and transversal plane loading (specifically abduction and internal rotation) strains the ACL more than either specific loading alone^8^, ^9^, ^10^; dynamic knee robustness in relation to ACL injury and re‐injury risk should optimally be evaluated under such conditions. An appropriate method may be to describe the 3D joint motion as an instantaneous rotation about an axis as performed using finite helical axis (FHA) methods.^11^, ^12^, ^13^ Such methods avoid the problem of movement cross‐over between sagittal, frontal, and transversal planes due to the independence of coordinate systems.^11^ Rotation of the FHA in relation to the flexion‐extension axis over consecutive rotation intervals describes how much knee kinematics diverge from sagittal plane motion. This presents a realistic measure of dynamic knee robustness in relation to ACL injury mechanics. FHA methods have shown to be sensitive in discriminating between tests with different knee joint demands,^12^ persons with a history of ACL injury (>20 years) from healthy‐knee controls,^13^ and different knee pathologies.^14^ Comprehensive information of the execution of challenging dynamic tasks (eg, hop landings) is gained when FHA methods are combined with inferential statistical methods for functional data. Functional data analysis (FDA) methods are increasingly used in biomechanics to present and compare kinematic and kinetic curves between groups; for instance, in the motor development of the vertical jump^15^ and in ACL injured versus controls during hopping.^16^, ^17^

Since a single training session with feedback may improve discrete measures of lower limb biomechanics among athletes,^18^ the question arises whether dynamic knee robustness is also improved. Utilizing both FHA and FDA methods answers whether dynamic knee robustness actually is superior in elite athletes than for non‐athletic persons when performing a sport‐mimicking task, or whether a superior task performance is mainly attributed to adopted movement strategies. Such knowledge is important to guide the decision‐making process in terms of neuromuscular training components to reduce the risk of knee injury and re‐injury among athletes and non‐athletes who aim to return to sport or physical activity. Our primary aim was thus to evaluate whether women elite athletes (ATH) display greater dynamic knee robustness and different trunk, hip, and knee landing mechanics than non‐athletic controls (CTRL) when performing a newly introduced and reliable one‐leg standardized rebound side hop task (SRSH).^19^ A secondary aim was to evaluate the knee function in these two groups (in terms of laxity, SRSH performances, maximal forward and vertical hop performances, and strength) in relation to the primary aim. We hypothesized that ATH would present greater dynamic knee robustness and larger trunk, hip, and knee flexion angles and moments than CTRL during landing, while also possessing superior knee function in terms of less laxity and better hop performances and strength.

## 2 MATERIALS AND METHODS

### 2.1 Participants

Participants were 39 physically active and healthy‐knee women (19 elite ATH, 20 CTRL) between 17 and 34 years of age. Exclusion criteria were any musculoskeletal or neurological pathology that might affect testing performance. One additional athlete was recruited to participate, but sustained an injury two days before planned testing. We focused solely on women since, relative to men; they have an approximately three times greater risk of sustaining an ACL injury^20^ and after ACL reconstruction show lower physical activity levels, poorer self‐estimated knee function scores, and lower rate of return to sport.^21^ ATHs (age: 21.0 ± 2.8 years; body height: 169 ± 5 cm; body mass: 64.2 ± 8.1) were competing in elite floorball and football teams in the highest or second highest division in the country (Tegner activity level score 8‐9). They had an International Physical Activity Questionnaire (IPAQ) median (range) total score of 4038 (5505) and a high activity median (range) score of 2400 (4320). To participate, ATHs had to regularly conduct knee‐specific training every week with the aim of improving lower limb control in multi‐directional movements, which they were to confirm both at recruitment and again prior to testing. Such exercises included lunges, jumps, and hops, side‐cutting movements with changes of direction, as well as agility and speed drills. CTRLs (age: 23.5 ± 3.5 years; body height: 169 ± 6 cm; body mass: 63.0 ± 5.8) were physically active but were not included if they participated in any more strenuous physical activity more than 4 days per week or performed knee‐specific training outside of gym or workout classes specifically aiming to improve dynamic knee control to decrease knee injury risk. This study design was adapted to clearly separate the groups. CTRLs had a Tegner activity level score median of 4 (range 2‐6; a 4 represents recreational sports of eg, running on uneven surface, cross‐country skiing, and aerobics), an IPAQ median (range) total score of 2484 (6576), and a high activity median (range) score of 1440 (2880). The study was approved by the regional ethical review board in Umeå, Sweden (Dnr. 2015/67‐31), and all participants provided written informed consent in agreement with the declaration of Helsinki before partaking.

### 2.2 Test procedure

All participants were tested at U‐motion lab, Umeå, Sweden. Participants first answered the Tegner activity scale and IPAQ and were then asked about any history of injuries undergoing a clinical knee examination by an experienced physiotherapist. Passive knee laxity (anterior translation) for both legs was assessed with a KT1000 arthrometer (Medmetric Corporation) using 30 lb of anterior pull force. Participants performed a series of low demanding tests (knee joint position sense, step down, two‐legged squat, and one‐leg balance) before the hop tests included in this study. The hop session started which included the maximal one‐leg hop for distance and vertical hop and then the SRSH test in focus, all described in detail below. Up to two practice trials were allowed for familiarization for all tests. Three to five trials were performed per leg for the maximal hops (a minimum of three successful trials were required but a maximum of five attempted trials were allowed to avoid fatigue) and 10 trials for SRSH. Participants started with the dominant leg (the preferred leg for kicking a ball) and alternated between legs for every trial (~5 seconds rest between trials). Participants performed all hop tests barefoot holding a rope (25 cm with knots) behind their back to emphasize the lower limb and avoid arm movements that would obscure the markers. Participants were instructed in all hop tests (except for the rebound landing in SRSH) to try to “stick” the landing and regain control as quickly as possible. For a trial to be deemed successful, the participants had to maintain a single leg stance for 3 seconds in the final position without putting the other foot down, making large adjustments with the standing leg to maintain stability, or letting go of the rope.

The maximal hop for distance was performed with participants standing upright on one leg and hopping forward as far as possible, landing on the same leg. The maximal vertical hop was then performed with the same starting position, by hopping as high as possible and landing on the same leg. Finally, the SRSH was performed for biomechanical analysis as previously described in detail.^19^ In short, participants hopped from one force plate to the side (laterally with respect to the hopping leg) over a distance normalized to 25% of body height onto another force plate (both masked by modular walkway elements), immediately rebounding back to their starting position. The first landing (after the lateral hop) was chosen for analysis and was defined from initial contact (IC) of the foot with the force plate (vertical force >20 N) until peak knee flexion. Contact time during this landing was defined from IC to takeoff in the rebound hop (vertical force <20 N).

Isometric peak knee extensor and flexor strength were tested using an isokinetic dynamometer (Kinetic communicator 125 Auto Positioning, The Chattanooga group inc) following the retailer's recommended settings with participants seated upright with a back angle of 78°, a seat bottom angle of 10°, the knee at ~65° (0° defined by the lever arm in a horizontal position), and secured using straps around the hip, both shoulders, and the thigh being tested. The 65° angle at the knee was chosen for maximal isometric strength output for both extensors and flexors.^22^ The dynamometer axis was aligned with the lateral femoral epicondyle and with the lowest part of the resistance pad placed ~10 mm proximal to the medial malleolus. A zero baseline correction was applied for each participant's leg before data collection. After a warm‐up of two trials of 2 seconds each with submaximal contraction, three maximal 5 seconds trials were conducted, with 5 seconds rest between repetitions. Knee extensors were tested first with participants maximally contracting their quadriceps by trying to extend their leg, followed by testing the knee flexors with participants maximally contracting their hamstrings by trying to bend their leg.

### 2.3 Data collection and analysis

A six degree‐of‐freedom model was constructed as previously described in detail.^19^ In short, 56 passive spherical markers (attached on the skin with double‐coated adhesive tape) and rigid clusters on thighs (four markers) were used to improve construct validity (reducing soft tissue artefacts) and increase reliability and precision of tracking.^23^ Hip joint centers were defined using a functional joint method (hip circumduction movement, pelvis as reference),^24^ while knee and ankle joint centers were defined from markers placed on the femur epicondyles and malleoli, respectively, during a stationary recording in standing. The same experienced test leader applied markers and instructed all participants.

Kinematic data during the hop tests were captured at 240 Hz using a motion capture system with eight cameras (Oqus 300, Qualisys AB) and were time synchronized with ground reaction force recordings (1200 Hz) from two Kistler force plates (model 9260AA, Kistler Instrument AG). The Qualisys Track Manager (v.2.2, Qualisys AB) and Visual3D (v.5.02.19, C‐Motion Inc) software were used for data processing and calculation. Joint moments normalized to body mass were calculated using inverse dynamics and described as external moments in this study, for example, an external knee flexion moment will tend to flex the knee. Kinematic and kinetic data were filtered with a fourth‐order bidirectional low‐pass Butterworth digital filter with a cutoff frequency of 15 Hz.

**Figure 1:**
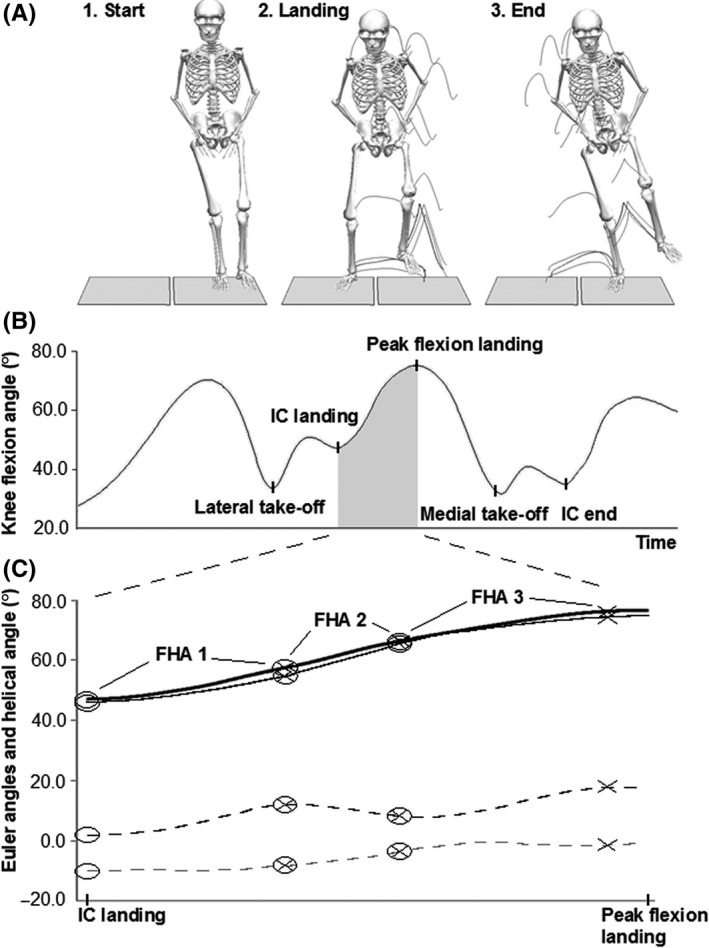
A schematic description of finite helical axis (FHA) inclination angle extraction for one trial. In (A) the SRSH is shown as performed on force plates for the right leg (with trail frames), with the person first hopping laterally and immediately hopping back to the initial position on the same leg. In (B) the right knee flexion angle curve is shown with the events marked. The landing phase of interest was defined from initial contact (IC) to peak knee flexion, as shown with the gray area. The knee motion curves are shown in (C) where the thick black line is the helical axis rotation, the thin black line is the Euler knee flexion/extension angle, the black dashed line is the Euler adduction/abduction angle, and the gray dashed line is the Euler internal/external rotation angle. Each ring indicates the start of knee helical motion for a new FHAs, and each cross indicates when 10° of helical rotation has occurred which generate the discrete FHA inclination angles. The more dissimilar the knee sagittal plane curve is to the helical axis curve, greater movement occur in frontal and/or transversal planes which generate greater inclination angles thus indicative of less knee robustness

Trunk, hip, and knee joint angles and moments were calculated using joint coordinate systems with the Cardan rotation sequence of *X* (mediolateral axis), *Y* (anteroposterior axis), and *Z* (longitudinal axis).^25^ Trunk angles were defined relative to the vertical axis of the laboratory coordinate system, hip joint angles from movement of the thigh relative to the pelvis, and knee joint angles from movement of the shank relative to the thigh. Curve data during the landing phase were extracted for angles in the sagittal and frontal planes for the trunk and for angles and moments in the sagittal, frontal, and transversal planes for the hip and knee joints. Dynamic knee robustness was evaluated using an FHA approach.^12^, ^13^, ^26^ This was done by first calculating the helical axis and the helical angle for the knee, where the helical axis defines knee motion direction and the helical angle defines the total angular rotation of the knee. For each 10° of helical rotation over the landing phase (from IC) of the SRSH (Figure 1), the knee motion direction was quantified by calculating the inclination between the helical axis and the flexion‐extension axis. The 10° interval was set to be as small as possible to be able to capture small movement changes (since ACL injuries often occur within the first 30‐50 ms),^3^, ^4^ but still to be within acceptable error levels (based on error simulations of FHA inclination angles). This computes how much the knee motion differs from strict flexion‐extension movement at certain motion intervals of the landing phase, regardless of whether this difference results from frontal or transversal plane movement. A lower inclination angle (close to 0) thus indicates greater dynamic knee robustness (less movement in frontal and/or transversal planes) while a greater inclination angle (close to 90°) implies that all knee movement occurs in frontal and/or transversal planes. Two examples of persons displaying low and high FHA inclination angles are found in the Figures S1 and S2.

Hop for distance length was calculated from the displacement of a marker on the foot between starting position to landing, and vertical hop height was calculated from the displacement of pelvis center of mass between standing to peak height. The single highest peak value for length and height from the successful hop trials completed was used in analyses. Dynamometer data (sampled at 1500 Hz) were filtered with a moving average of 60 ms and normalized to body mass, with the single highest peak value from the three trials completed used for analyses.

### 2.4 Statistical analysis

Outcome variables for the dominant leg were used for between‐group comparisons. The mean values for FHA inclination angles and their time occurrences from IC and the mean motion curves of the first five successful trials for each person were calculated. The successive FHAs were numbered FHA‐1, FHA‐2, FHA‐3, etc, each representing a knee helical rotation interval of 10°. Since each trial may result in a different number of FHAs (depending on range of knee motion), at least three of the five trials had to generate the same specific FHA (eg, FHA‐2) to be included in the analyses (this range of trials did not influence the FHA inclination angles). Thus, FHAs provided by two or less trials were not considered representative for that person's knee movement and were excluded from analyses. Since ACL injury mechanics have been estimated to occur as fast as 30‐50 ms after impact,^3^, ^4^ the initial FHA‐1 inclination angle was considered most important and thus evaluated for trial‐to‐trial reliability. An intraclass correlation coefficients model_(3,5)_ were used and showed excellent reliability with a coefficient of 0.77. FHA inclination angles were analyzed between groups using independent *t* tests. Knee function outcomes were analyzed with a MANOVA and followed with Bonferroni post‐hoc tests to correct for multiple comparisons. Partial eta^2^ as effect sizes (ES, 0.01 = small, 0.1 = medium, 0.25 = large)^27^ were presented.

The kinematic and kinetic mean curves (representing the individuals) were analyzed with a FDA method for testing the equality of the two mean functions of two functional populations. The curves were aligned within the landing phase to account for individual differences in time between the aforementioned landing events. Prior to the analysis, the landing phase was discretized in 51 points. This adjustment enabled continuous data series to be compared between persons and groups using identical relative time points. The curves were analyzed between ATH and CTRL by applying a functional *t* test, based on the interval‐wise testing procedure.^28^ Such an approach enabled the identification of time intervals where ATH and CTRL differed. The unadjusted *P*‐values correspond to a point‐wise control of the probability of wrongly detecting a significant difference. The interval‐wise testing‐adjusted *P*‐values ensured that the probability of wrongly rejecting *any interval* (ie, false positive) was below the chosen significance level, within each analysis. All computations and statistical analyses were conducted using the Statistical Package for the Social Sciences (v.23, IBM SPSS Statistics) and R (v.3.2.0), with level of significance being 0.05.

## 3 RESULTS

**Figure 2:**
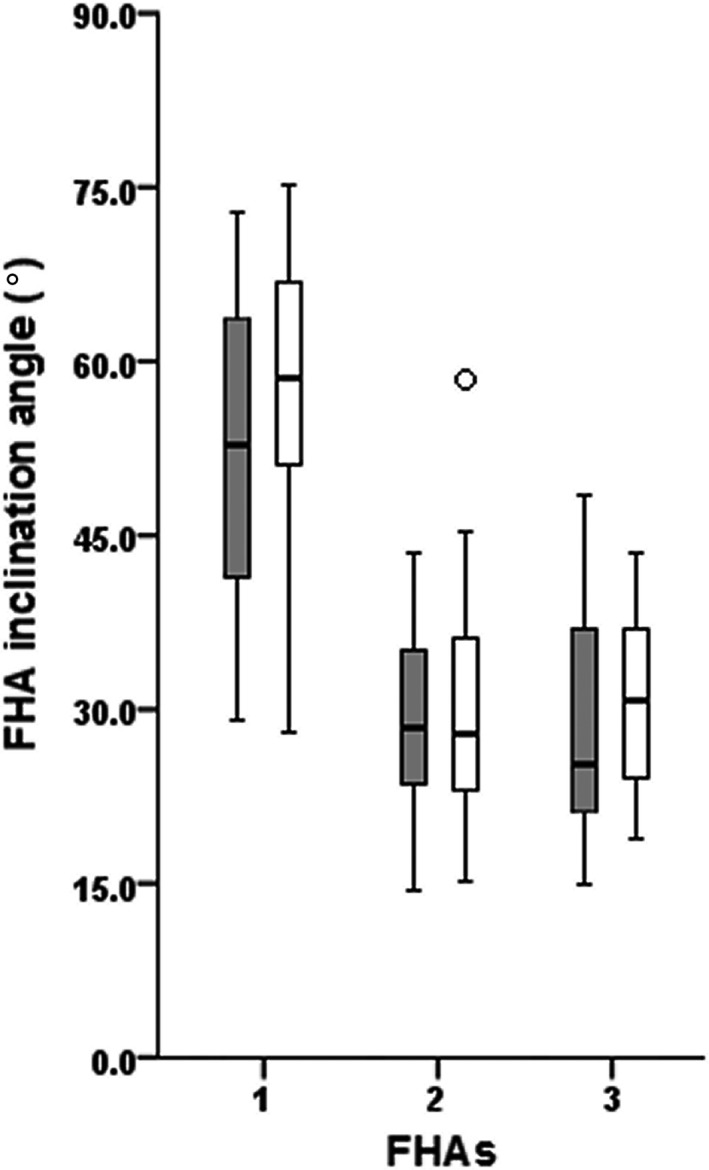
Finite helical axis (FHA) inclination angles for FHA‐1—FHA‐3 during the landing phase. No significant differences in dynamic knee robustness were shown between ATH (gray boxes) and CTRL (white boxes). One outlier among CTRL for FHA‐2 (small circle) due to a continued (see FHA‐1) large movement in both frontal and transversal planes relative the sagittal plane. Each FHA inclination angle represents a knee helical motion of ~10°

Elite athletes and CTRL had no significant difference in range of knee motion, and hence, an equal amount of FHAs were generated in the groups. The number of participants that provided FHA‐1—FHA‐5 occurrences was 19, 17, 16, 6, and 1, respectively, for ATH and 20, 20, 18, 11, and 0, respectively, for CTRL. ATH had a mean occurrence of 3.1 FHAs (SD: 1.0) and CTRL 3.5 FHAs (0.7) during the landing phase (non‐significant, *P* = 0.213, low ES). Further statistical analyses between groups were therefore performed for inclination angles of FHA‐1—FHA‐3, with no significant differences found (*P* ≥ 0.150, low ESs, Figure 2). The times (in seconds) after IC that FHA‐1—FHA‐3 occurred for ATH and CTRL were as follows: 0.066 (0.017) and 0.063 (0.011), respectively, for FHA‐1, 0.105 (0.018) and 0.105 (0.014), respectively, for FHA‐2, and 0.151 (0.030) and 0.150 (0.027), respectively, for FHA‐3.

**Figure 3:**
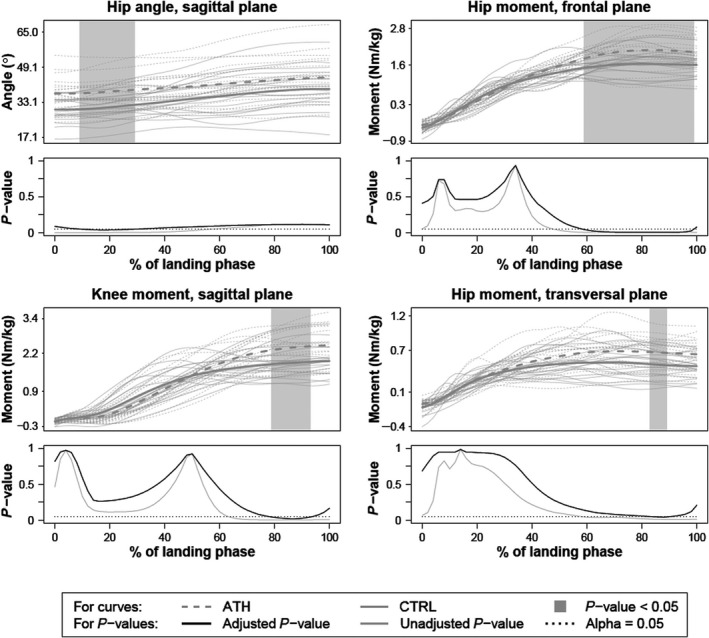
Curves of angles and moments that were significantly different between groups during the first landing phase. Hip flexion (+)/extension (−) angle presented in top left, hip adduction (+)/abduction (−) moment in top right, knee flexion (+)/extension (−) moment in bottom left, and hip internal (+)/external (−) rotation moment in bottom right. The thick dashed and solid gray lines correspond to group means and the thin gray lines to individuals. The gray areas within the plots indicate significant between‐group differences detected using functional *t* tests at a 5% level. These *P*‐values are shown as curves beneath each plot with the interval‐wise testing‐adjusted *P*‐value in black solid line and the unadjusted *P*‐value in gray solid line. The horizontal dashed line indicates the 5% level of significance

Regarding the kinematic curves, only hip flexion angle was significantly different between groups (Figure 3). Greater flexion angles (37‐38° versus 30‐31°) were observed in ATH between 9% and 29% of the landing phase, although immediately at IC the unadjusted *P*‐value identified a significant difference. For the kinetic curves, significantly higher moments were detected for ATH for hip adduction between 59% and 99%, hip internal rotation between 83% and 89%, and knee flexion between 79% and 93% of the landing phase (Figure 3). These moments were ~ 1.2‐1.4 times greater than those of CTRLs throughout the identified time intervals of significant differences. (All analyzed kinematic and kinetic curves are found in Figure S3).

Elite athletes displayed better knee function (main effect: *P*‐value = 0.007, large ES) as indicated by significantly less passive laxity, longer maximal one‐leg hop for distance, greater number of successful hops in SRSH, shorter contact time in rebound in SRSH, and greater knee extensor strength than CTRLs (Table 1). ATHs also presented tendencies to greater maximal one‐leg vertical hop height and knee flexor strength (9%‐11% larger) nearing the statistical significance threshold. Low to moderate *r* 2 values (0.10‐0.39) were found between strength and hop performances within each group, thus indicating different aspects of functional performance.

**Table 1:** Knee function outcomes of the dominant leg of women athletes and controls

	ATH (n = 19)	CTRL (n = 20)	*P*‐value (ES)	
	Mean (SD)	Mean (SD)	Main effect	Post‐hoc*
Passive anterior knee laxity 30 Ib (mm)	5.6 (2.1)	7.3 (1.6)	**0.007 (0.45)**	**0.009 (0.18)**
Maximal one‐leg hop for distance (m)	1.34 (0.17)	1.13 (0.18)		**0.001 (0.28)**
Maximal one‐leg vertical hop (m)	0.24 (0.03)	0.22 (0.03)		0.077 (0.08)
Successful hops SRSH (No. out of 10)	9.1 (1.1)	7.9 (1.8)		**0.037 (0.12)**
Contact time in Rebound (s)	0.41 (0.14)	0.70 (0.32)		**0.001 (0.26)**
Peak knee extensor torque (N m/kg)	2.71 (0.44)	2.23 (0.57)		**0.007 (0.19)**
Peak knee flexor torque (N m/kg)	1.20 (0.22)	1.08 (0.22)		0.097 (0.07)

Abbreviation: ATH, elite athletes; CTRL, controls; ES, effect size; SD, standard deviation; SRSH, standardized rebound side hop.

Bold *P*‐values indicate a significant multivariate main effect or significant univariate effects at 0.05 level.

* Adjustment for multiple comparisons using Bonferroni post‐hoc correction.

## 4 DISCUSSION

The major finding of this study is that elite women athletes who display superior knee function (less laxity, better hop performances, and quadriceps strength) than non‐athletic controls still present similar dynamic knee robustness (similar FHA inclination angles) as the controls during sport‐mimicking SRSH landings. This is true despite that athletes demonstrated different movement strategies with larger hip flexion angles, hip frontal and transversal plane moments, and knee flexion moments, during specific intervals of the landing phase. Such results indicate that elite athletic training may result in a greater ability to keep the knee robust while performing the task more efficiently by increased engagement of the hip.

In sports in which considerable loads are placed on the knee joint, athletes struggle with demands of keeping joint mobility while still maintaining joint stability. This constitutes a challenge since sports performance and biomechanical risk factors for injury are closely related.^7^ Previous statements therefore recommend athletes to regularly train for and learn proper movement techniques (reduce knee motion in the frontal and transversal planes), while simultaneously improving agility and speed during dynamically challenging tasks.^6^, ^7^ Indeed, such training has proven successful in decreasing injury occurrence^29^, ^30^ and improving lower limb landing mechanics (injury prevention perspective).^31^, ^32^ We calculated a quantified measure of dynamic knee robustness based on FHA inclination angles relative to the knee flexion‐extension axis during consecutive motion intervals of 10°. We evaluated this measure during the critical landing phase where most knee injuries and specifically ACL tears occur.^3^, ^4^, ^5^ Contrary to our hypothesis, the similar and substantial FHA‐1 inclination angle for ATH and CTRL shows that the greatest knee motion occurred in the frontal and/or transverse planes during the first 10° of knee helical motion (cf Figure 2). These results may, however, be explained to some extent by the differences in task performance. ATHs greater moments of hip adduction, hip internal rotation, and knee flexion during the landing (~20%‐40% higher) in relation to their shorter contact times (~40% lower) implicates higher demands of knee function relative to CTRLs. ATHs may therefore have an ability to retain the same level of dynamic knee robustness while demonstrating superior hop performance, including better stretch‐shortening cycle capacities. Nevertheless, we argue that it would be more optimal if ATHs could also improve our measure of robustness by decreasing FHA‐1 inclination angles to reduce knee motion related to knee injury. The short average time event for FHA‐1 (63‐66 ms, both groups) is comparable to the time intervals reported in the literature of 30‐50 ms in which most ACL injuries are believed to occur.^3^, ^4^ It could therefore be argued that it is highly important to also focus on movement strategy preparation *before* IC rather than only focus on correct lower limb mechanics *during* landing, to avoid a high FHA‐1 inclination angle.

In assessing an athlete's readiness to return to sport after an ACL injury with or without reconstructive surgery, the affected leg is usually compared to the contralateral unaffected leg. Such an approach may overestimate the function of the affected knee due to decreased neuromuscular function of both affected and non‐affected legs.^33^ Healthy‐knee controls not subjected to proper training may also lack the appropriate level of knee function to act as a reference to the affected leg of athletes. The elite athletes in this study (floorball, football) that reported to routinely perform knee‐specific training were targeted as references of optimal knee function and robustness. The few existing biomechanical studies that have compared athletes and non‐athletic controls in a context relevant to our results show that athletes present shorter contact times, higher average and peak vertical forces, and lower sagittal angular displacements at the whole kinematic chain (hip, knee, and ankle joints) when performing an 80 cm drop jump (seven athletes and 11 physically active controls, sex unspecified).^34^ Further results show less anterior knee laxity under passive and active (muscle contracted) conditions^35^; and greater ankle plantarflexion, although no differences in hip and knee joint flexion angles, during a bilateral drop landing task (four athletes and four controls, all women).^36^ Our results corroborate these findings of shorter contact time and less knee laxity in ATH versus CTRL. The lack of hip and knee kinematic differences between groups for these studies, in relation to our findings, may be explained by task specificity and small sample sizes.

One strategy applied by ATHs was to have greater hip flexion at and early after IC (Figure 3). Such a strategy lowers the center of mass to increase movement control and is commonly performed as part of training in team sport athletes to enable faster changes of directions, for instance. Even though ATHs displayed higher hip moments in frontal and transversal planes, they had similar hip and knee angle curves in these motion planes to CTRLs. These results indicate better capabilities of landing control since greater effort is needed to withstand such motions. In relation to the significant association between restricted hip rotation and increased risk of ACL injury^37^ and the significant positive relation between hip adduction and (ACL injury prone) knee abduction movement,^38^ hip joint mechanics deserve special attention. A knee joint specific focus is, however, also necessary to induce the desired alterations in knee joint landing mechanics.^32^ Further research is required to investigate how athletes may improve measures of dynamic knee robustness, such as FHA inclination angles or similar measures, during sport‐mimicking tasks including cutting maneuvers and our SRSH task.

Some methodological aspects of this study need consideration. Using the knee helical motion, interval of 10° to provide the FHA inclination angles is considered a strength since it evaluates the relative knee motion between the motion planes, which is relevant for non‐contact knee injuries such as ACL injury.^8^, ^9^, ^10^ If we instead would have used knee flexion intervals of 10° or specific time intervals, we might miss important spatial information due to a loss in the relation between knee movement planes. Consequences include a decreased representation of dynamic knee stability and robustness with relevance to non‐contact knee injuries. However, we acknowledge that other methods to quantify dynamic knee stability and robustness are available and may show different results than ours, although this requires further research. Further, the knee flexion angle at IC varied between participants (although similar between groups) but seem not to have an effect on inclination angles for FHA‐1 to FHA‐3 due to non‐significant correlations (*r* ≤ 0.287, *P* ≥ 0.099). Another strength is the use of FDA, with the adjusted *P*‐values that ensures the identification of time intervals (in comparison with commonly used discrete values) where the groups differed within the chosen significance level. Our focus on women also limits the generalization of our results to women only, since men may have different landing mechanics^39^ and injury risk factors.^20^ Lastly, studies that use motion capture systems with skin markers are always faced with soft tissue artefacts, which we tried to limit with the use of rigid clusters on the thighs to increase construct validity of data^23^ and by having a standardized test protocol with the same test leader applying markers on all test sessions. The helical axis/angle also avoids the sequence dependency encountered by Cardan/Euler angles that may introduce errors in frontal and transversal planes. The SRSH is also a reliable test specifically designed to evaluate angles and moments, with reported within‐session intraclass correlation coefficients of 0.95‐0.97 for knee abduction and internal rotation angles at initial contact and for peak angles.^19^

## 5 PERSPECTIVES

Dynamic knee robustness and proper movement control are important to decrease ACL injury incidence and risk of re‐injury incidence.^40^ We used FHA measures to quantify dynamic knee robustness during a challenging sports‐mimicking task, addressing possible differences between groups of women elite athletes and non‐athletic but physically active controls. Differences in movement strategies observed as landing mechanics were found between the athletes (who displayed superior knee function in terms of less laxity, greater hop performances, and strength) and the controls, primarily at the hip and in knee joint flexion moment, while the dynamic knee robustness measures were comparable. Our results challenge the notion that high‐level athletes (with reported elements of knee‐specific neuromuscular training) display greater dynamic knee robustness (evaluated with FHA inclination angles) than active non‐athletes. Elite athletes may therefore direct attention to improve both dynamic knee robustness and movement strategies during sport‐mimicking tasks, both in preparation for impact and during landing, which may decrease their risk of ACL injury.

## CONFLICT OF INTEREST

The authors declare that they have no conflict of interest.

## ETHICAL APPROVAL

The Regional Ethical Review board in Umeå approved the study (reference: 2015/67‐31).
