# Supplementary figures and images for: Dynamic knee control and movement strategies in athletes and non‐athletes in side hops: Implications for knee injury
Source: Scand J Med Sci Sports. 2019 Apr 25;29(8):1181–9. doi: 10.1111/sms.13432 (PMC6850355; doi:10.1111/sms.13432)

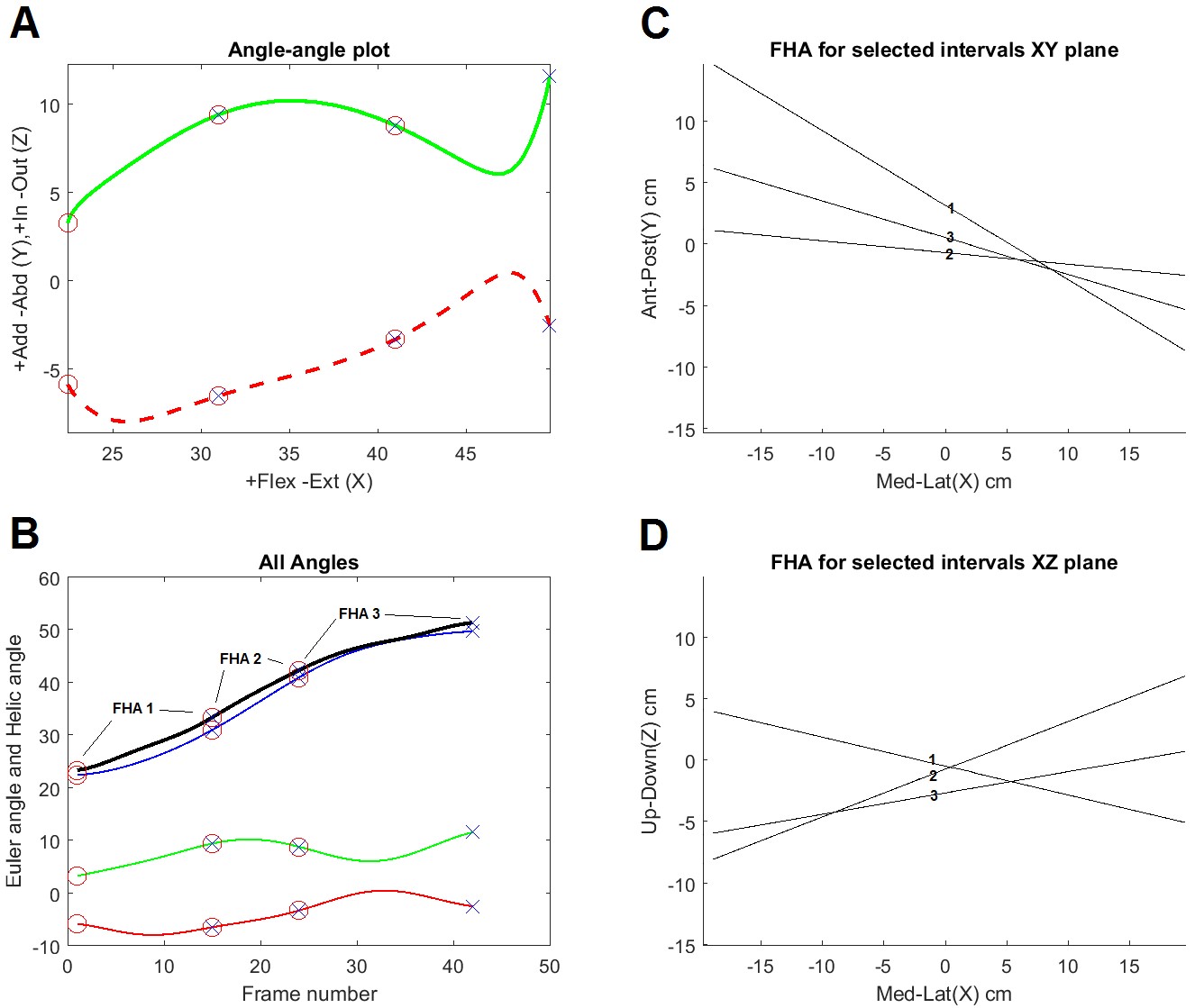

Supplement: Supplementary file 1 [file SMS-29-1181-s001.jpg]

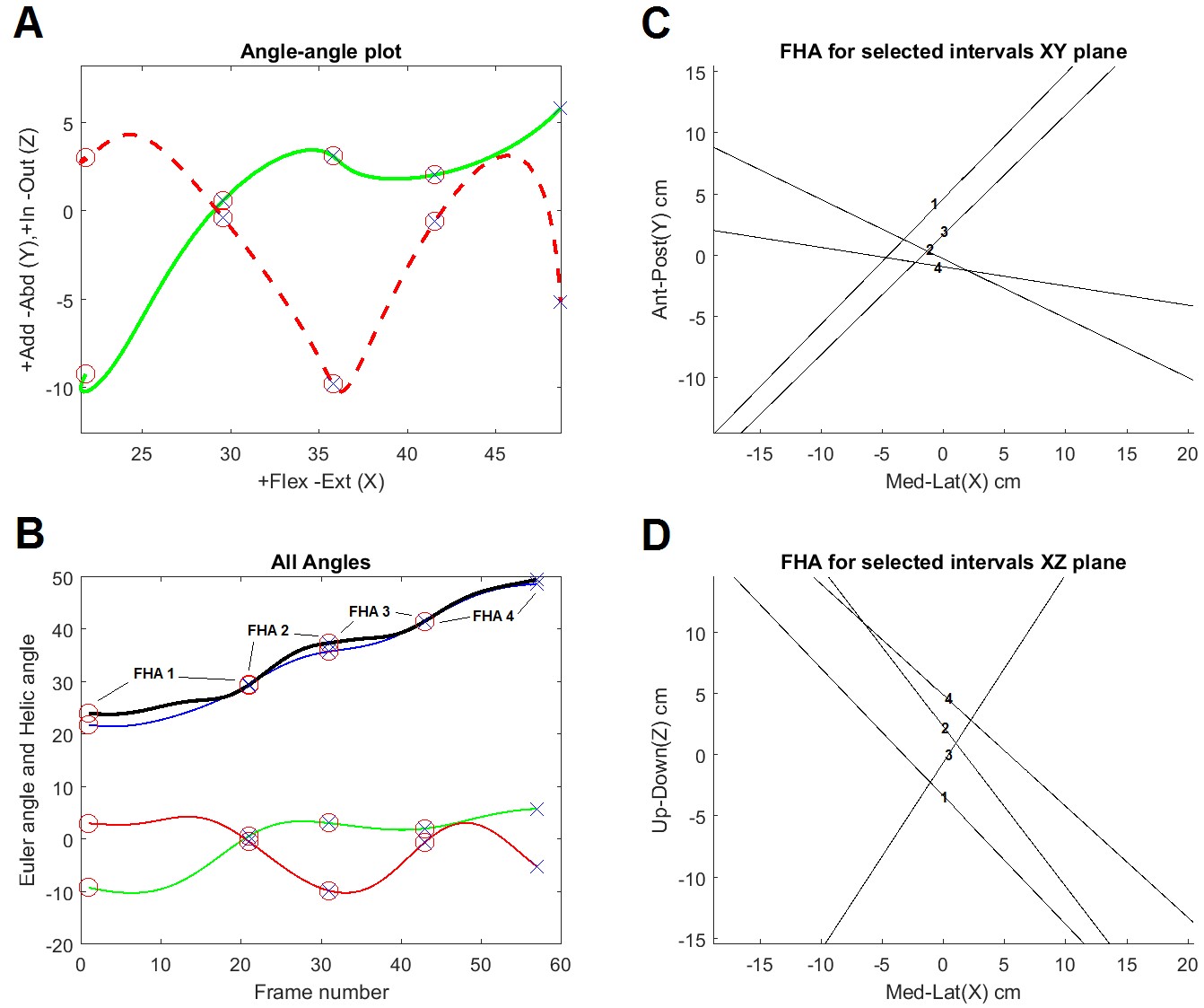

Supplement: Supplementary file 2 [file SMS-29-1181-s002.jpg]

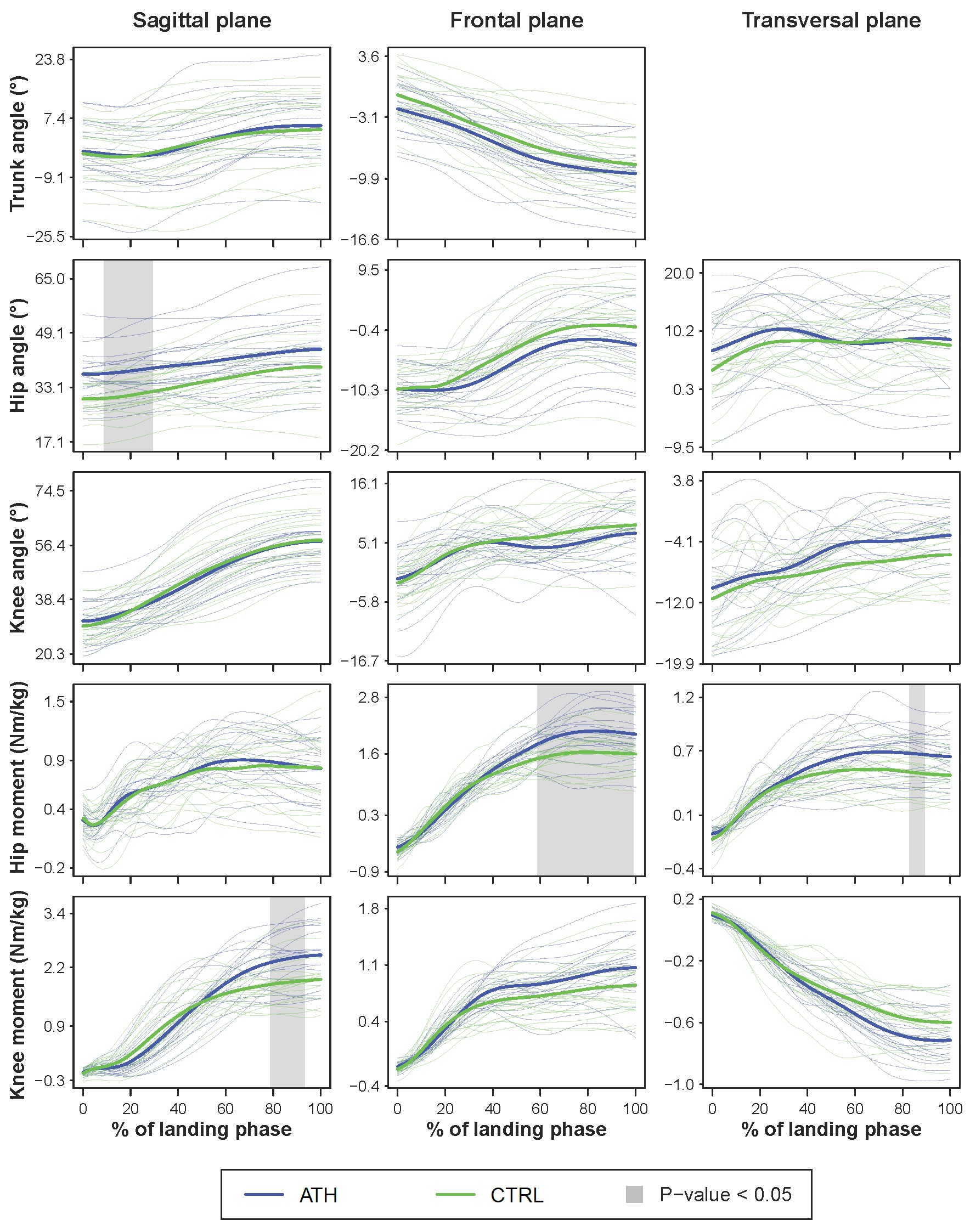

Supplement: Supplementary file 3 [file SMS-29-1181-s003.jpg]
