# Supplementary material for: Dynamic knee control and movement strategies in athletes and non‐athletes in side hops: Implications for knee injury
Source: Scand J Med Sci Sports. 2019 Apr 25;29(8):1181–9. doi: 10.1111/sms.13432 (PMC6850355; doi:10.1111/sms.13432)
Supplement: Supplementary file 4 [file SMS-29-1181-s004.docx]

Supplementary files for review

**Figure S1 Title.** An example of a hop landing trial from a person that displays low FHA inclination angles.

**Figure S1 Legend.** A. The knee Euler angle curves for adduction/abduction (green line) and internal/external rotation (red dashed line) planes relative to flexion/extension (X-axis). B. Knee motion curves during the landing. The thick black line is the helical axis rotation, the blue line is the Euler flexion/extension angle, the green line is the Euler adduction/abduction angle, and the red line is the Euler internal/external rotation angle. For both A and B, rings indicate the start of the knee helical motion for a new FHAs, and crosses indicate when 10˚ of helical rotation has occurred which generate the discrete FHA inclination angles. The more dissimilar the knee sagittal plane curve is to the helical axis curve, the greater movement occurs in the frontal and/or transversal planes. This generates greater inclination angles thus indicative of less knee robustness. C and D. The FHA inclination angles displayed in XY plane (viewed from above) and XZ plane (viewed posteriorly) respectively show how the helical axes are rotated in relation to the knee flexion-extension axis (X-axis). The FHA inclination angles are: 35.8˚ for FHA-1, 14.2˚ for FHA-2 and 19.1˚ for FHA-3, respectively.

**Figure S2 Title.** Example of a hop landing trial from a person that displays high FHA inclination angles.

**Figure S2 Legend.** A. The knee Euler angle curves for adduction/abduction (green line) and internal/external rotation (red dashed line) planes relative to flexion/extension (X-axis). B. Knee motion curves during the landing. The thick black line is the helical axis rotation, the blue line is the Euler flexion/extension angle, the green line is the Euler adduction/abduction angle, and the red line is the Euler internal/external rotation angle. For both A and B, rings indicate the start of the knee helical motion for a new FHAs, and crosses indicate when 10˚ of helical rotation has occurred which generate the discrete FHA inclination angles. The more dissimilar the knee sagittal plane curve is to the helical axis curve, the greater movement occurs in the frontal and/or transversal planes. This generates greater inclination angles thus indicative of less knee robustness. C and D. The FHA inclination angles displayed in XY plane (viewed from above) and XZ plane (viewed posteriorly) respectively show how the helical axes are rotated in relation to the knee flexion-extension axis (X-axis). The FHA inclination angles for this hop landing trial are: 55.7˚ for FHA-1, 54.3˚ for FHA-2, 61.2˚ for FHA-3 and 42.7˚ for FHA-4, respectively.

**Figure S3 Title.** Curves of angles and moments during the first landing phase for all variables analyzed.

**Figure S3 Legend.** The thick blue and green lines correspond to group means and the thin lines to individuals. The gray areas within the plots indicate significant between-group differences detected using functional t-tests at a 5% level. Flexion, adduction and internal rotation defined as positive, while extension, trunk lateral bending, abduction and external rotation defined as negative.
